# 

**Published:** 1908-12-15

**Authors:** 


CONTENTS

Event and Comment - - - - - - - - 595

Observations on the Cause and Prevention of Dental Caries,
*By J. Sim Wallace, M.D.,D.Sc.Glasg., L.D.S., R. C.S. Eng.* 599

Medical and Dental Examination of School Children,
*By Thomas B. Cooley, M.D.* 614

Indents - - - - - - - - - - 626

Announcements - - - - - - - - - 637

